# Penicillin-Binding Protein Transpeptidase Signatures for Tracking and Predicting β-Lactam Resistance Levels in *Streptococcus pneumoniae*

**DOI:** 10.1128/mBio.00756-16

**Published:** 2016-06-14

**Authors:** Yuan Li, Benjamin J. Metcalf, Sopio Chochua, Zhongya Li, Robert E. Gertz, Hollis Walker, Paulina A. Hawkins, Theresa Tran, Cynthia G. Whitney, Lesley McGee, Bernard W. Beall

**Affiliations:** Respiratory Diseases Branch, Division of Bacterial Diseases, National Center for Immunization and Respiratory Diseases, Centers for Disease Control and Prevention, U.S. Department of Health and Human Services, Atlanta, Georgia, USA

## Abstract

β-Lactam antibiotics are the drugs of choice to treat pneumococcal infections. The spread of β-lactam-resistant pneumococci is a major concern in choosing an effective therapy for patients. Systematically tracking β-lactam resistance could benefit disease surveillance. Here we developed a classification system in which a pneumococcal isolate is assigned to a “PBP type” based on sequence signatures in the transpeptidase domains (TPDs) of the three critical penicillin-binding proteins (PBPs), PBP1a, PBP2b, and PBP2x. We identified 307 unique PBP types from 2,528 invasive pneumococcal isolates, which had known MICs to six β-lactams based on broth microdilution. We found that increased β-lactam MICs strongly correlated with PBP types containing divergent TPD sequences. The PBP type explained 94 to 99% of variation in MICs both before and after accounting for genomic backgrounds defined by multilocus sequence typing, indicating that genomic backgrounds made little independent contribution to β-lactam MICs at the population level. We further developed and evaluated predictive models of MICs based on PBP type. Compared to microdilution MICs, MICs predicted by PBP type showed essential agreement (MICs agree within 1 dilution) of >98%, category agreement (interpretive results agree) of >94%, a major discrepancy (sensitive isolate predicted as resistant) rate of <3%, and a very major discrepancy (resistant isolate predicted as sensitive) rate of <2% for all six β-lactams. Thus, the PBP transpeptidase signatures are robust indicators of MICs to different β-lactam antibiotics in clinical pneumococcal isolates and serve as an accurate alternative to phenotypic susceptibility testing.

## IMPORTANCE

The human pathogen *Streptococcus pneumoniae* is a leading cause of morbidity and mortality worldwide. β-Lactam antibiotics such as penicillin and ceftriaxone are the drugs of choice to treat pneumococcal infections. Some pneumococcal strains have developed β-lactam resistance through altering their penicillin-binding proteins (PBPs) and have become a major concern in choosing effective patient therapy. To systematically track and predict β-lactam resistance, we obtained the sequence signatures of PBPs from a large collection of clinical pneumococcal isolates using whole-genome sequencing data and found that these “PBP types” were predictive of resistance levels. Our findings can benefit the current era of strain surveillance when whole-genome sequencing data often lacks detailed resistance information. Using PBP positions that we found are always substituted within highly resistant strains may lead to further refinements. Sequence-based predictions are accurate and may lead to the ability to extract critical resistance information from nonculturable clinical specimens.

## INTRODUCTION

*Streptococcus pneumoniae* (pneumococcus) is an important human pathogen causing large numbers of cases of pneumonia, bacteremia, and meningitis globally. Penicillin and other β-lactam antibiotics have been the primary means of treating pneumococcal infections for decades. The first detection of a pneumococcus with reduced penicillin susceptibility (MIC ≥ 0.12 µg/ml) occurred in 1967 in Australia (1); in the United States, penicillin-nonsusceptible pneumococci became an emerging problem during the 1990s (2–7). Introduction of pneumococcal conjugated vaccines (PCVs), which target 7 to 13 of the more than 90 known pneumococcal serotypes, was associated with a decrease in resistant pneumococcal infections (8–10). Nonetheless, emergence and spread of β-lactam resistance, particularly among serotypes not targeted by PCVs, remains a major concern. Systematically tracking β-lactam resistance is critical for both disease surveillance and choosing effective therapy for patients. A penicillin MIC of 0.12 µg/ml is considered resistant for pneumococcal meningitis, while for nonmeningitis disease, a 16-fold increased MIC for this antibiotic is considered susceptible. There is an extraordinary range of β-lactam resistance expressed by different clinically relevant pneumococcal strains. For these reasons, it is critical to have the capability to accurately predict a wide range of MICs for β-lactam antibiotics.

In clinical isolates, β-lactam resistance is primarily driven by alterations in the transpeptidase domains (TPDs) of penicillin-binding proteins (PBPs) that reduce affinity for the antibiotics to attach to these sites. Growth inhibition of wild-type pneumococcal strains by most β-lactam antibiotics is primarily due to inhibition of PBP2x (11). The primary role of PBP1a, PBP2b, and PBP2x for determining β-lactam MICs was indicated when a mixture of three alleles from a single highly penicillin-resistant pneumococcal strain was used to transform a susceptible strain to achieve the same level of resistance (12). Published evidence supports that changes within PBP2b and PBP2x are essential for lower-level β-lactam resistance (13–15), with these and additional changes within PBP1a essential for high-level resistance (16, 17). Other changes both within and outside the PBPs presumably compensate for fitness costs (18–20). Many resistant *pbp* alleles enter into the pneumococcal population through interspecies recombination with other *mitis* group members of the *Streptococcus* genus (21–23). Subsequent intraspecies recombination and mutation further diversify *pbp* loci, resulting in a large number of *pbp* alleles associated with a broad range of β-lactam MICs.

To effectively track genotypes and their associated MICs of β-lactam-nonsusceptible pneumococci, we propose a classification system in which a pneumococcal isolate is assigned a “PBP type” based on amino acid residues in the TPDs of the three major PBPs, PBP1a, PBP2b, and PBP2x. Here we report 307 PBP types identified from 2,528 invasive *S. pneumoniae* isolates and use the PBP type to predict β-lactam MICs as measured by microdilution. Our results indicate that the PBP typing system will provide an alternative to conventional susceptibility testing and might improve our ability to extract critical resistance information from nonculturable clinical specimens.

## RESULTS

### Increased β-lactam MIC associated with PBP types containing divergent TPD variants.

**FIG 1:**
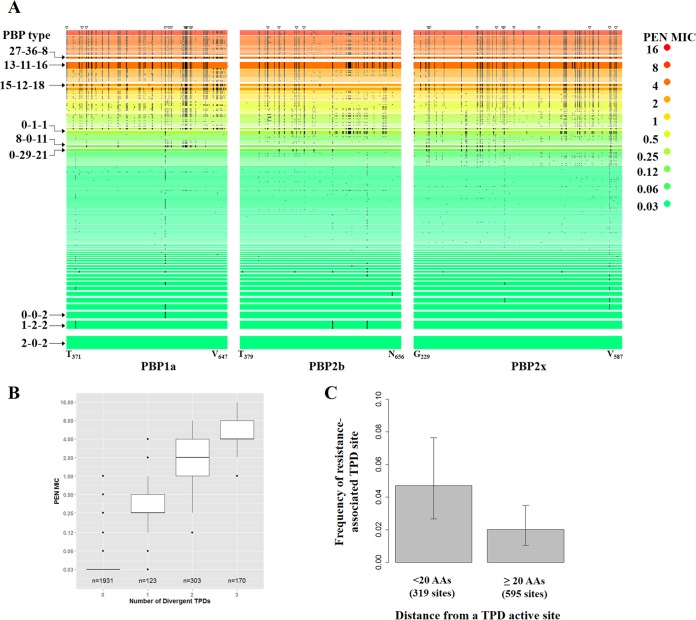
Amino acid changes in penicillin-binding protein (PBP) transpeptidase domains (TPDs) associated with increased MICs. (A) Each row is a unique PBP type, and a black bar indicates that the aligned amino acid differed from the amino acid in the reference PBP type 2-0-2. The width of the row is proportional to the number of isolates. Rows were sorted from top to bottom by decreasing order of median penicillin (PEN) MICs (in micrograms per milliliter) and then by increasing order of number of isolates. The PBP type 2-0-2 row also shows the start and end amino acid position of each TPD. There were 27 TPD sites in which an amino acid differed from that in PBP type 2-0-2 in all isolates with PEN MICs of ≥4 µg/ml (open triangles at the top of the figure); we define these 27 TPD sites as resistance-associated TPD sites. (B) Boxplot of PEN MICs among isolates containing 0, 1, 2, or 3 divergent TPDs. A divergent TPD showed less than 90% amino acid sequence identity with the corresponding TPD in PBP type 2-0-2. Whiskers indicate the farthest value that is within 1.5 interquartile range (IQR) of the hinges. (C) Resistance-associated TPD sites were more frequently found among amino acid sites that are close to an active site (<20 amino acids [AAs]). The three TPD domains contain a total of 914 amino acid sites, which were classified into two groups according to whether the distance to an active site is less than 20 AAs (*n* = 319) or more than 20 AAs (*n* = 595). The frequency of resistance-associated TPD sites in each group is shown. Error bars are 95% CIs.

From the 2,528 *S. pneumoniae* isolates studied here, we identified 68, 78, and 118 unique TPD amino acid sequences for PBP1a, PBP2b, and PBP2x, respectively. We observed 307 unique combinations of these sequences which defined the PBP types. The 2,528 isolates exhibited a wide range of β-lactam MICs (Table 1) as measured by microdilution. Figure 1A compares the median penicillin (PEN) MICs across all 307 PBP types. Typical PBP types associated with PEN-susceptible isolates were 2-0-2 (*n* = 248), 1-2-2 (*n =* 162), and 0-0-2 (*n* = 134) (PBP type naming system explained in “Isolates and Characterization” in Materials and Methods). PBP types with intermediate PEN resistance (median MIC, 0.12 to 1 µg/ml) included 0-1-1 (*n* = 61), 8-0-11 (*n* = 44), and 0-29-11 (*n* = 56). Common PBP types with the median PEN MIC of ≥2 µg/ml were 13-11-16 (*n =* 121), 15-12-18 (*n =* 53), and 27-36-8 (*n =* 42). PEN-resistant PBP types tended to show 30 or more amino acid changes in the three TPDs compared to the susceptible PBP type 2-0-2 (Fig. 1A). In fact, there was a strong positive correlation between the number of amino acid changes and PEN MIC (ρ = 0.82 [see Fig. S1 in the supplemental material]).

**TABLE 1:** Distribution of β-lactam MICs in the study sample according to broth microdilution testing

Antibiotic^a^	Parameter^b^	Value for parameter^c^										
PEN	MIC (µg/ml)	≤0.03	0.06	0.12	0.25	0.5	1	2	4	8	16	NA
	No. of isolates	1,702	93	110	121	61	61	140	178	60	1	1
AMO	MIC (µg/ml)	≤0.03	0.06	0.12	0.25	0.5	1	2	4	8	>8	NA
	No. of isolates	1,745	171	84	31	50	67	105	83	155	22	15
MER	MIC (µg/ml)		≤0.06	0.12	0.25	0.5	1	>1				NA
	No. of isolates		1,942	67	46	129	232	28				84
TAX	MIC (µg/ml)		≤0.06	0.12	0.25	0.5	1	2	4	8	16	NA
	No. of isolates		1,840	120	64	67	180	148	16	26	3	64
CFT	MIC (µg/ml)					≤0.5	1	2	4	8		NA
	No. of isolates					1618	141	86	17	6		660
CFX	MIC (µg/ml)					≤0.5	1	2	>2			NA
	No. of isolates					2,040	24	40	417			7

a Abbreviations: PEN, penicillin; AMO, amoxicillin; MER, meropenem; TAX, cefotaxime; CFT, ceftriaxone; CFX, cefuroxime.

b The MIC and the number of isolates with the indicated MIC for each antibiotic are shown.

c NA, no MIC data available.

We classified a TPD variant as “divergent” if it showed less than 90% amino acid sequence identity with the corresponding TPD in PBP type 2-0-2. Thus, an isolate could harbor 0 to 3 divergent TPDs in PBP1a, PBP2b, and PBP2x. We observed a stepwise increase in PEN MIC as the number of divergent TPDs in an isolate increased (Fig. 1B). While almost all PEN-susceptible isolates contained no divergent TPDs, isolates containing 1, 2, and 3 divergent TPDs showed median PEN MICs of 0.25 µg/ml, 2 µg/ml, and 4 µg/ml, respectively. The differences in median PEN MICs were highly significant (*P* < 1 × 10^−15^ for all pair-wise comparisons by Mann-Whitney U test). Similar stepwise increases in MICs with increasing numbers of divergent TPDs were also observed for the other five β-lactams (see Fig. S2 in the supplemental material).

Amino acid changes common to all highly resistant isolates (PEN MIC of ≥4 µg/ml) appeared to be distributed unevenly across the TPDs. There were 27 TPD positions in which an amino acid change relative to PBP type 2-0-2 was found in 100% (*n =* 253) of highly resistant isolates (Fig. 1A; see Table S1 in the supplemental material). We observed a modestly yet significantly higher density of such positions within 20 amino acids (AA) from a TPD active site (Fig. 1C, 0.047 versus 0.021, *P* = 0.038, Fisher’s exact test). While some of the 27 positions have been implicated in altering affinity to β-lactams, such as T371 and TSQF574– 577 in PBP1a (24), T446 and E476 in PBP2b (25), and T338 in PBP2x (26), many others were of unclear function.

### The PBP type correlated with β-lactam MICs across diverse MLSTs.

**FIG 2:**
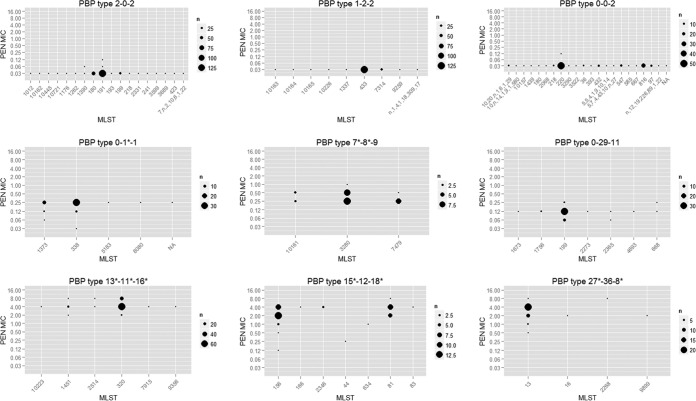
Distribution of PEN MICs across MLSTs within nine representative PBP types; in most PBP types, the MICs cluster around ±1 dilution. Divergent TPDs, which showed less than 90% amino acid sequence identity with the corresponding TPD in PBP type 2-0-2, are indicated with an asterisk (e.g., 0-1*-1).

Our study sample was comprised of isolates with diverse genomic backgrounds, including 403 different multilocus sequence types (MLSTs). The PBP type and MLST were correlated but not completely overlapping (see Fig. S3 in the supplemental material), which allowed us to examine the relative contribution of PBP and genomic background to β-lactam MIC. First, we examined the distribution of PEN MICs across MLSTs within 9 representative PBP types (Fig. 2), of which 3 showed median PEN of 0.03 µg/ml (top panel), 3 showed median PEN of 0.12 to 0.25 µg/ml (middle panel), and another 3 showed median PEN of 2 to 4 µg/ml (bottom panel). The number of MLSTs observed within each PBP type ranged from 2 to 18. We found essentially identical MICs across MLSTs in the susceptible PBP types. For example, 244 of the 248 PBP type 2-0-2 isolates were of the same PEN MIC even though they represented 18 different MLSTs. The resistant PBP types showed slightly more MIC variation (Fig. 2, middle and bottom panels), but no association between MLST and PEN MIC level (treated as a categorical variable) within a PBP type was found (*P* > 0.05 by Fisher’s exact test). Similar within-PBP type MIC distributions were also observed for the other 5 antibiotics (see Fig. S4 in the supplemental material). The results suggested that PBP type consistently correlated with β-lactam MIC across diverse MLST-defined genomic backgrounds.

The above notion was further supported by an analysis of variance using mixed-effect models (Table 2). While the MLST-only model explained 73% to 91% of variations in the log_2_ MIC for the 6 β-lactams (Table 2, model 1), the PBP type-only model explained a higher proportion (94% to 99%) of the variations. In the model incorporating both the PBP type and the MLST (Table 2, model 3), the amount of variation attributed to the PBP type remained around 94% to 98%, but the amount of variation attributed to the MLST dropped to 0.00% to 0.95%. Compared to the PBP type-only model, adding the MLST improved goodness of fit minimally and only for 3 of the 6 β-lactams (Table 2, model 3 versus model 2). The results indicated that after accounting for the PBP type, genomic background contributed little to β-lactam MICs at the population level.

**TABLE 2:** Analysis of variation in log_2_ (MIC) incorporating random effects for the PBP type and the MLST for the three models evaluated^a^

Antibiotic^b^	Variation^c^ explained by:				*P* value^d^	
	Model 1 (MLST only)	Model 2 (PBP type only)	Model 3			
			MLST	PBP type	Model 3 vs model 1	Model 3 vs model 2
PEN	91.3	97.9	0.06	97.8	<2 × 10^−16^	0.10
AMO	91.0	98.7	0.02	98.6	<2 × 10^−16^	0.32
MER	90.4	97.5	0.24	97.3	<2 × 10^−16^	1 × 10^−8^
TAX	89.1	97.9	0.09	97.9	<2 × 10^−16^	0.005
CFT	73.3	94.2	0.00	94.2	<2 × 10^−16^	1
CFX	90.6	98.1	0.95	97.3	<2 × 10^−16^	4 × 10^−14^

a Models were constructed with the log_2_-transformed MIC as the dependent variable. Model 1 included only multilocus sequence type (MLST) as the covariate. Model 2 included only PBP type as the covariate. Model 3 used both PBP type and MLST as covariates. These models incorporated random effect(s) for all covariate(s). The only fixed-effect term was the intercept.

b Abbreviations: PEN, penicillin; AMO, amoxicillin; MER, meropenem; TAX, cefotaxime; CFT, ceftriaxone; CFX, cefuroxime.

c Percentage of variance that is attributed to the indicated model covariate(s).

d The *P* value of the likelihood ratio test.

### PBP type as β-lactam MIC predictor.

**FIG 3:**
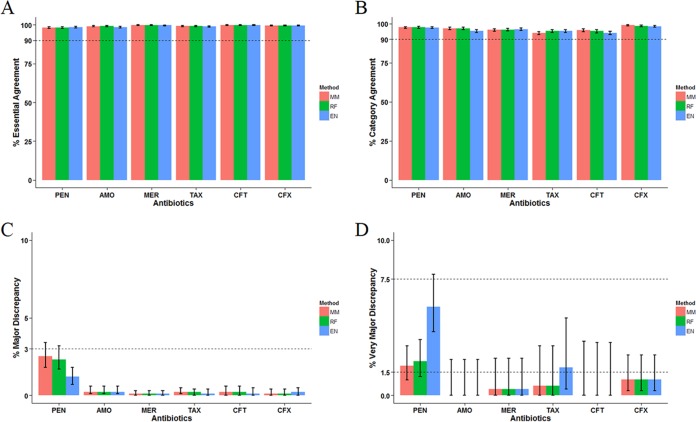
Agreement between the predicted MIC and the microdilution MIC among isolates of trained PBP types. The mode MIC (MM) model assigned the most frequently seen MIC of a trained PBP type to a test isolate of the same PBP type. The random forest (RF) and elastic net (EN) models were designed to quantify the contribution of each individual TPD position from the training data set and combine these contributions to make a prediction. See Materials and Methods for detailed model description. The percent essential agreement (A), category agreement (B), major discrepancy (C), and very major discrepancy (D) were calculated for six antibiotics. Error bars are 95% CIs.

The predominant role of PBP type in explaining MIC variation motivated us to test whether PBP type, or its corresponding TPD amino acid sequences, could be used as a practical predictor of β-lactam MICs. We constructed three predictive models as described in Materials and Methods. The predicted MICs of the six β-lactams generated by the leave-one-out approach were compared against the microdilution MICs (Fig. 3). In isolates of trained PBP types (trained PBP type explained in “Predictive models of β-lactam MIC” in Materials and Methods), MICs predicted by the mode MIC (MM) model showed essential agreement (EA) of >98%, category agreement (CA) of >94%, major discrepancy (maj) rate of <3%, lower 95% confidence interval (95% CI) of the very major discrepancy (vmj) rate of <1.5%, and upper 95% CI of the vmj rate of <7.5% for all six β-lactams (Fig. 3). Similar results were observed for the random forest (RF) and elastic net (EN) models (Fig. 3), except that the EN model showed a higher vmj rate for PEN than the other two models did (Fig. 3D). These results supported the predicted MIC by the MM and RF models as an acceptable equivalent to the microdilution MIC among the trained PBP types. Predicted PEN MICs by the MM model based on all isolates are shown in Table S2 in the supplemental material. Figure S5 shows a diagram indicating how to predict MICs for a specific isolate using these tables.

**FIG 4:**
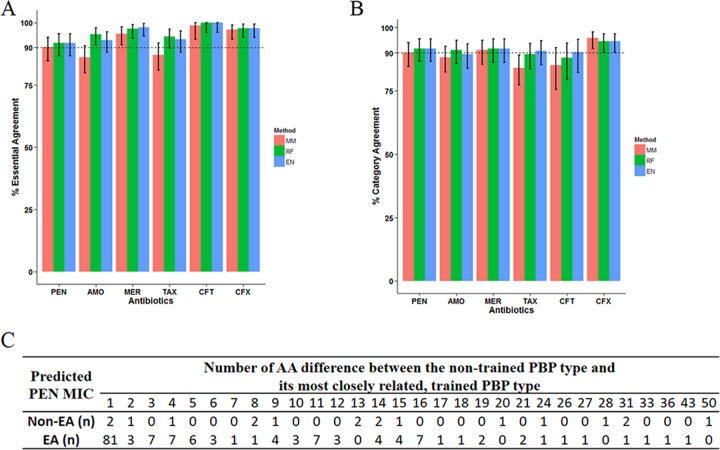
Agreement between the predicted MIC and the microdilution MIC among isolates of nontrained PBP types. The mode MIC (MM) model approximated a nontrained PBP type by the most closely related trained PBP type and assigned the most frequently seen MIC of the trained PBP type to the test isolate. For the random forest (RF) and elastic net (EN) models, any amino acid not seen in the training data set was approximated by a corresponding training amino acid with the least BLOSUM62 distance. See Materials ad Methods for detailed model description. (A and B) The rates of essential agreement (A) and category agreement (B) were calculated for the six antibiotics. Error bars are 95% CIs. (C) Relationship between the number of amino acid (AA) differences and outcome of EA for PEN MIC (in micrograms per milliliter) predicted by the MM method.

For isolates of nontrained PBP types (nontrained PBP type explained in “Predictive models of β-lactam MIC” in Materials and Methods), a generally lower percentage of EA and CA was observed for predictions made by the three models (Fig. 4A and B). The RF and EN models appeared to perform slightly better in EA than the MM model (Fig. 4A). The maj and vmj errors were also higher among nontrained PBP types (see Fig. S6 in the supplemental material), but the evaluation was limited by the small sample size, which resulted in wide confidence intervals. For PEN, a nontrained PBP type differed from its most closely related, trained PBP type by 1 to 50 AA with a median of 2 AAs (Fig. 4C). Among the 83 isolates whose nontrained PBP type showed only 1 AA difference from its most closely related, trained PBP type, the proportion EA for PEN predicted by the MM model (81/83) was not significantly different from what was observed among isolates of trained PBP type (2,314/2,355, *P* = 0.66 by Fisher’s exact test). For every 1 additional AA difference, there was, on average, a 1.09-fold (95% CI, 1.14 to 1.04) decrease in the odds of being EA [logistic regression, *t*(170) = −3.69 and *P* = 0.0002]. Thus, the predictive performance of the MM model decreases significantly for the nontrained PBP types containing more than 1 AA difference.

**FIG 5:**
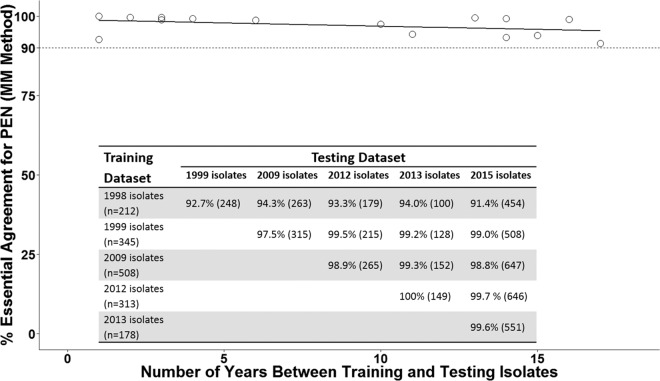
Regression line showing the effect of time between the training and testing data sets on percent essential agreement (EA). Isolates in one of the surveillance years 1998, 1999, 2009, 2012, and 2013 were used as the training data set for the MM model to predict PEN MIC for isolates in subsequent years with trained PBP types. The inset table shows percent EA (number of isolates used to calculate EA) in the indicated testing data set. Based on data in the table, the year-specific percent EA was plotted against separation time between the training and testing data sets (open circles). A fitted linear regression line is shown (solid line).

Because the pneumococcal genomic background evolves over time, we examined whether the MM model prediction performance could be affected by the length of time between the training data set and the testing data set. Isolates in one of the five surveillance years (1998, 1999, 2009, 2012, and 2013) were used as the training data set for the MM model, and the fitted model was used to predict PEN MIC for isolates in subsequent years with trained PBP types. EA between the predicted PEN MIC and microdilution MIC was calculated for each year tested (Fig. 5). The year-specific EA ranged from 91.4% to 100% with a median of 98.9% (Fig. 5). We found no significant association between separation time and year-specific percentage EA [Fig. 5, linear regression, *t*(13) = −1.59 and *P* = 0.14].

## DISCUSSION

In this study, we proposed a PBP typing system that links amino acid sequence variation in the TPDs of PBP1a, PBP2b, and PBP2x to β-lactam MIC levels among invasive pneumococcal isolates. We found that PBP types containing divergent TPD variants—those exhibiting more than 10% amino acid sequence difference from the typical susceptible PBP type 2-0-2—were associated with increased β-lactam MICs. Isolates with high-level PEN resistance commonly harbored a divergent TPD within all three PBPs. One possible reason is that epistasis between *pbp* loci is essential for these strains to maintain both high-level β-lactam resistance and fitness. The TPD positions in which amino acids frequently differed between susceptible and resistant isolates appeared to be distributed nonrandomly. This may reflect structural constraints dictating that only changes in specific TPD positions would allow a viable, resistant TPD variant. Alternatively, this could indicate that the observed resistant TPD sequences have not yet diverged sufficiently from recent founder sequences. Additional studies are needed to understand the relative contributions of these mechanisms.

In genomically tracking β-lactam resistance, a typing system that correlates with the phenotype closely and robustly is desirable. Since sequence variations both within and outside the PBPs have been reported to influence β-lactam MICs (19, 20, 27), we examined how much the phenotype could vary among isolates of the same PBP type but of diverse genomic backgrounds as defined by MLST. The MICs within susceptible PBP types were very stable regardless of genomic background. The results were consistent with the notion that sequence variations outside the three TPDs may alter the level of resistance in isolates carrying resistant PBPs, but these variations rarely confer resistance themselves (28). While the resistant PBP types tended to show larger MIC variations, there was no evidence that the MIC differed significantly by MLST within the same PBP type. Several different factors could explain the lack of association between MLST and MIC within a PBP type. One possibility is that MLST failed to represent the non-TPD variations actually modulating MICs. Another possibility is that non-TPD variations influence MIC only minimally so that there was not enough power to detect such effects. Nonetheless, analysis of variance showed that the PBP type, when incorporated into a model for random effect, explained the vast majority of variation in β-lactam MICs both before and after accounting for MLST, suggesting that the PBP type was a dominant and robust determinant of β-lactam MIC at the population level.

One practical application of our proposed PBP typing system is to predict the β-lactam resistance level based on sequence information. This application could be important in developing susceptibility testing for clinical specimens from which an isolate cannot be recovered. It may also facilitate estimating the prevalence and spread of β-lactam resistance in the current era when bacterial whole-genome sequencing data rapidly accumulate, yet accompanying MIC information is not always available or reliable. Here we developed three basic models to predict β-lactam MICs based on PBP type. The simplest MM model just assigned the most frequently seen MIC of a trained PBP type to a test isolate of the same PBP type and approximated any nontrained PBP type by the most closely related trained PBP type. The more-complex RF and EN models were designed to quantify the contribution of each individual TPD position from the training data set and combine these contributions to make a prediction. Predicted MICs by MM and RF models showed essentially equivalence with the microdilution MIC for trained PBP types but not for the nontrained ones. The results supported the use of these predicted MICs as valid susceptibility testing results for isolates of trained PBP types. It is also important to obtain microdilution MICs for all newly found PBP types, thus expanding the number of trained PBP types. Better modeling design and more training data could help identify conserved amino acid sites that are predictive of MICs in other data sets. Characterizing the specific contributions of substitutions at these conserved, predictive sites is important because the pneumococcal genome is highly plastic, precluding the documentation of all possible PBP types. Further, models that quantify the contributions of individual TPD positions and/or their interactions to β-lactam MICs may provide insights into resistance mechanisms and should be a priority of future investigation.

## MATERIALS AND METHODS

### Isolates and characterization.

The study sample was 2,528 broadly representative pneumococcal isolates selected from the Active Bacterial Core surveillance (ABCs) over the years 1998 to 2015. ABCs is an active, population-based and laboratory-based surveillance system that is part of the Centers for Disease Control and Prevention’s (CDC) Emerging Infections Program. Cases of invasive pneumococcal diseases (IPD) were defined by the isolation of pneumococci from a normally sterile site in residents of the surveillance areas in 10 different states (29–31) (see ABCs surveillance reports for population sizes, IPD incidence, antimicrobial susceptibility data, and other information at http://www.cdc.gov/abcs/reports-findings/surv-reports.html).

Isolates were characterized by a combination of conventional testing and short-read whole-genome sequence (WGS) analysis. MICs for six β-lactam antibiotics, penicillin (PEN), amoxicillin (AMO), meropenem (MER), cefotaxime (TAX), ceftriaxone (CFT), and cefuroxime (CFX), were determined by the broth microdilution method as previously described (32). MIC interpretive standards are shown in Table S3 in the supplemental material and are consistent with CLSI document M100-23 (33). Unless otherwise specified, PEN susceptibility and resistance refer to PEN MIC of ≤0.06 and ≥0.12 µg/ml, respectively. When MIC was analyzed as a numeric variable, an MIC of “= *X*” was treated as value *X*; an MIC of “≤*X*” was approximated as value *X*; and an MIC of “>*X*” was approximated as value 2*X*.

Multilocus sequence types (MLSTs) and the TPD amino acid sequences of PBP1a, PBP2b, and PBP2x were extracted using a validated pneumococcal typing pipeline as described previously (34). Databases of 68, 78, and 118 unique TPD amino acid sequences were compiled for PBP1a, PBP2b, and PBP2x, respectively (see Table S4 in the supplemental material). Each unique TPD amino acid sequence was assigned an identifier, and the three-number combination from each isolate was assigned as its “PBP type.” For example, PBP type 2-0-2 describes an isolate containing a composite TPD amino acid sequence pattern of PBP1a-2, PBP2b-0, and PBP2x-2. All isolates through 2013 were serotyped with latex agglutination and the quellung reaction employing CDC antisera. Serotypes of isolates recovered after 2013 were determined by WGS and the typing pipeline (34).

PBP active site motifs (amino acid sequences) were defined as the following according to Hakenbeck et al. (20): S_370_TMK, S_466_SN, and K_557_TG in PBP1a, S_386_TMK, S_443_SN, and K_614_TG in PBP2b, and S_337_TMK, S_395_SN, and K_547_SG in PBP2x. The position of a motif’s first residue was used in calculating distance from an active site.

### Analysis of variance for β-lactam MIC.

Mixed-effect models incorporating random effects for PBP type and MLST were used for the analysis of variance. We chose mixed-effect models because the observed PBP types and MLSTs represented a sample (subset) of all possible types in the pneumococcal population. Additionally, mixed-effect models could facilitate the analysis of data that were unbalanced and contained cells of 0 count in a contingency table, which was the case for our study sample.

For each β-lactam, four models were constructed with the log_2-_transformed MIC as the dependent variable and using another or other factors or variables as the covariate(s). In model 1, only MLST was used as a covariate. In model 2, only PBP type was used as a covariate. In model 3, both PBP type and MLST were used as covariates. In model 4, PBP type, MLST, and their interaction term were used as covariates. These models incorporated random effect(s) for all covariate(s). The only fixed-effect term was the intercept. Nested models were compared by a likelihood ratio test; a *P* value of <0.05 was considered evidence of significantly better fit for the more-complex model. The R package “lme4” was used for model fitting and comparison. Model 4 showed no significant increase in goodness of fit compared to model 3 for any β-lactams and therefore was excluded from further analysis.

### Predictive models of β-lactam MIC.

The study sample (2,528 pneumococcal isolates) was divided into a training data set and a test data set. MICs in the test data set were predicted using models parameterized by the training data set. We denoted a PBP type as “trained” if it was present in the training data set with MIC data; otherwise, the PBP type was denoted as “nontrained.” In the “leave-one-out” cross-validation, which represents a special case of dividing the study sample into training and test data sets, each isolate was used in turn as the testing data set, while all other isolates were used as the training data set.

Three predictive models were evaluated: (i) mode MIC (MM) model, (ii) random forest (RF) model, and (iii) elastic net (EN) model.

### (i) MM model.

In the mode MIC model, the highest MIC among the most frequently observed MIC(s) for a PBP type in the training data set was assigned as the predicted MIC of the same PBP type in the test data set. Any PBP type not seen in the training data set was approximated by a training PBP type that showed the highest amino acid identity.

### (ii) RF model.

In the random forest model, we used the amino acid at each position of the three TPDs as predictors to train an RF model for the continuous outcome log_2_ (MIC). The trained model then predicted the MIC of a test isolate based on its TPD amino acid sequence. For a given position in the TPDs, any amino acid not seen in the training data set was approximated by the training amino acid with the least BLOSUM62 distance. The R package “randomForest” was used for RF model training and prediction.

### (iii) EN model.

Similar to the RF model, the amino acid at each position of the three TPDs was used as predictors to train an elastic net model for the continuous outcome log_2_ (MIC). The R package “glmnet” was used for EN model training and prediction.

To evaluate prediction performance, we calculated the essential agreement (EA), category agreement (CA), very major discrepancy (vmj), and major discrepancy (maj) between the predicted MIC (new method) and broth microdilution MIC (reference method) according to the FDA guidance document for antimicrobial susceptibility test systems (35). Briefly, the FDA guidance document’s definitions follow: CA, agreement of interpretive results (susceptible [S], intermediate [I], or resistant [R]); EA, agreement within (plus or minus) one twofold dilution of the reference MIC; maj, the reference category result is S and the new method result is R; vmj, the reference category result is R and the new method result is S. Criteria for acceptable performance in the FDA guidance document include (i) essential and category agreement of >89.9%; (ii) a maj rate of ≤3%; and (iii) an upper 95% confidence limit for the true vmj rate of ≤7.5% and the lower 95% confidence limit for the true vmj rate of ≤1.5%.

### Statistics.

Correlation between two numerical variables was quantified by Spearman’s rank correlation coefficient (ρ). Association between two categorical variables was evaluated by Fisher’s exact test. Equal median between two groups was examined by Mann-Whitney U test. Confidence interval for proportion was constructed using the exact binomial method. All statistical analyses were performed in R version 3.2.2 (36); graphics were also created in R version 3.2.2.

## SUPPLEMENTAL MATERIAL

Figure S1  Positive correlation between the number of amino acid (AA) differences from PBP type 2-0-2 and PEN MIC. Download Figure S1, TIF file, 0.2 MB

Figure S2  Boxplot of PEN MIC among isolates containing 0, 1, 2, or 3 divergent TPDs. A divergent TPD was defined as less than 90% amino acid sequence identity with the corresponding TPD in PBP type 2-0-2. Whiskers indicate the farthest value that is within 1.5 interquartile range (IQR) of the hinges. Download Figure S2, TIF file, 0.2 MB

Figure S3  Distribution of isolates by PBP type and MLST. Each dot represents an isolate(s) with the same PBP type and MLST. The size of the dot is proportional to the number of isolates. Download Figure S3, TIF file, 0.2 MB

Figure S4  Distribution of MICs across MLSTs within nine representative PBP types for AMO (A), MER (B), TAX (C), CFT (D), and CFX (E). Download Figure S4, TIF file, 0.5 MB

Figure S5  Diagram showing how to predict MICs for a specific isolate using the PBP sequence database (Table S4) and the PBP type to MIC tables (Table S2). Download Figure S5, TIF file, 0.1 MB

Figure S6  Agreement between the predicted MIC and the microdilution MIC among nontrained PBP types. See Materials and Methods for detailed model descriptions. (A and B) The rates of major discrepancy (A) and very major discrepancy (B) were calculated for the six antibiotics. Error bars are 95% confidence intervals. Download Figure S6, TIF file, 0.1 MB

Table S1  27 TPD positions in which an amino acid change relative to PBP type 2-0-2 was found all highly resistant isolates (PEN MIC ≥4 µg/ml). Table S1, XLSX file, 0.01 MB

Table S2  Penicillin (PEN) MICs predicted by the PBP type using the MM model. Table S2, XLSX file, 0.02 MB

Table S3  Interpretive standards for β-lactam antibiotics. Table S3, XLSX file, 0.01 MB

Table S4  Databases of 68, 78, and 118 unique TPD amino acid sequences were compiled for PBP1a, PBP2b, and PBP2x, respectively. Table S4, XLSX file, 0.02 MB
