# Supplementary figures and images for: Penicillin-Binding Protein Transpeptidase Signatures for Tracking and Predicting β-Lactam Resistance Levels in Streptococcus pneumoniae
Source: mBio. 2016 Jun 14;7(3):e00756-16. doi: 10.1128/mBio.00756-16 (PMC4916381; doi:10.1128/mBio.00756-16)

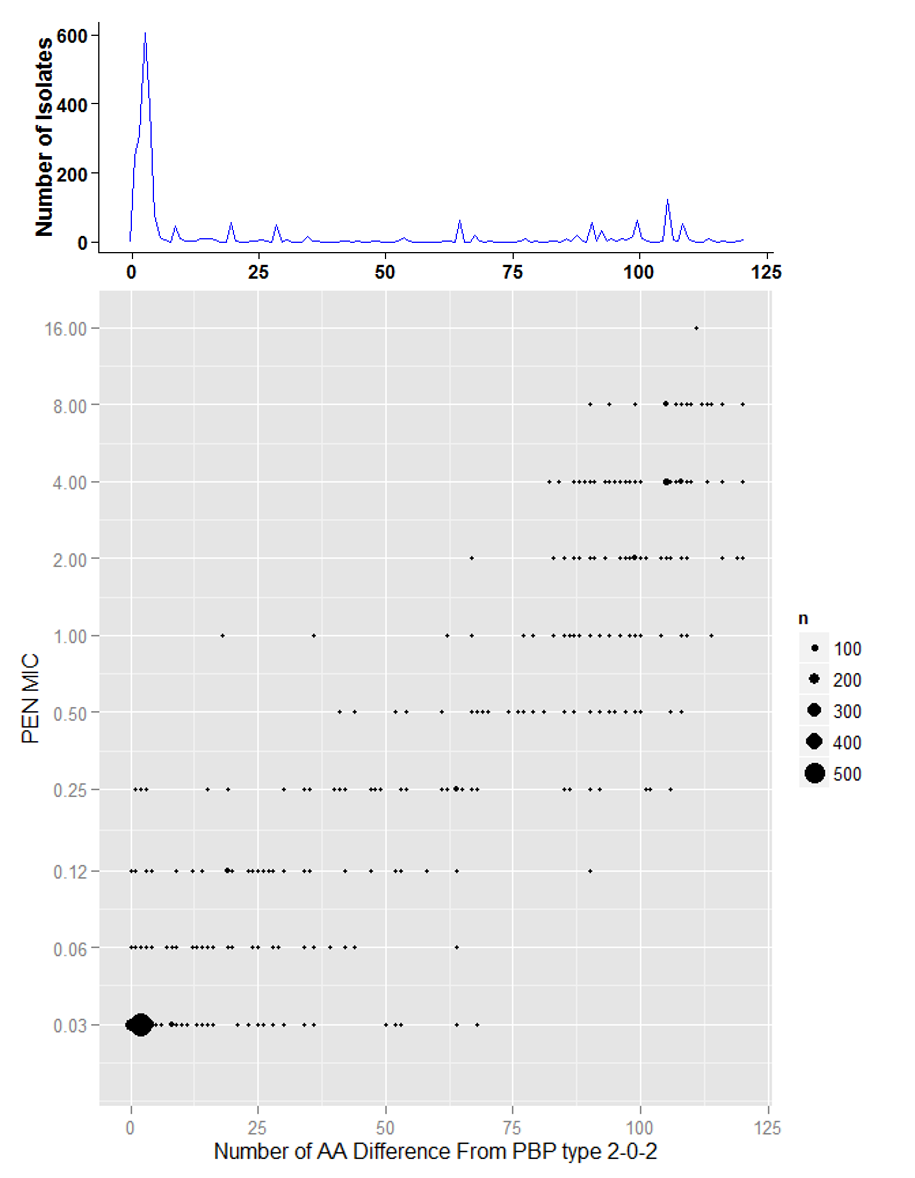

Supplement: Figure S1 — Positive correlation between the number of amino acid (AA) differences from PBP type 2-0-2 and PEN MIC. Download [file mbo004142843sf1.tif]

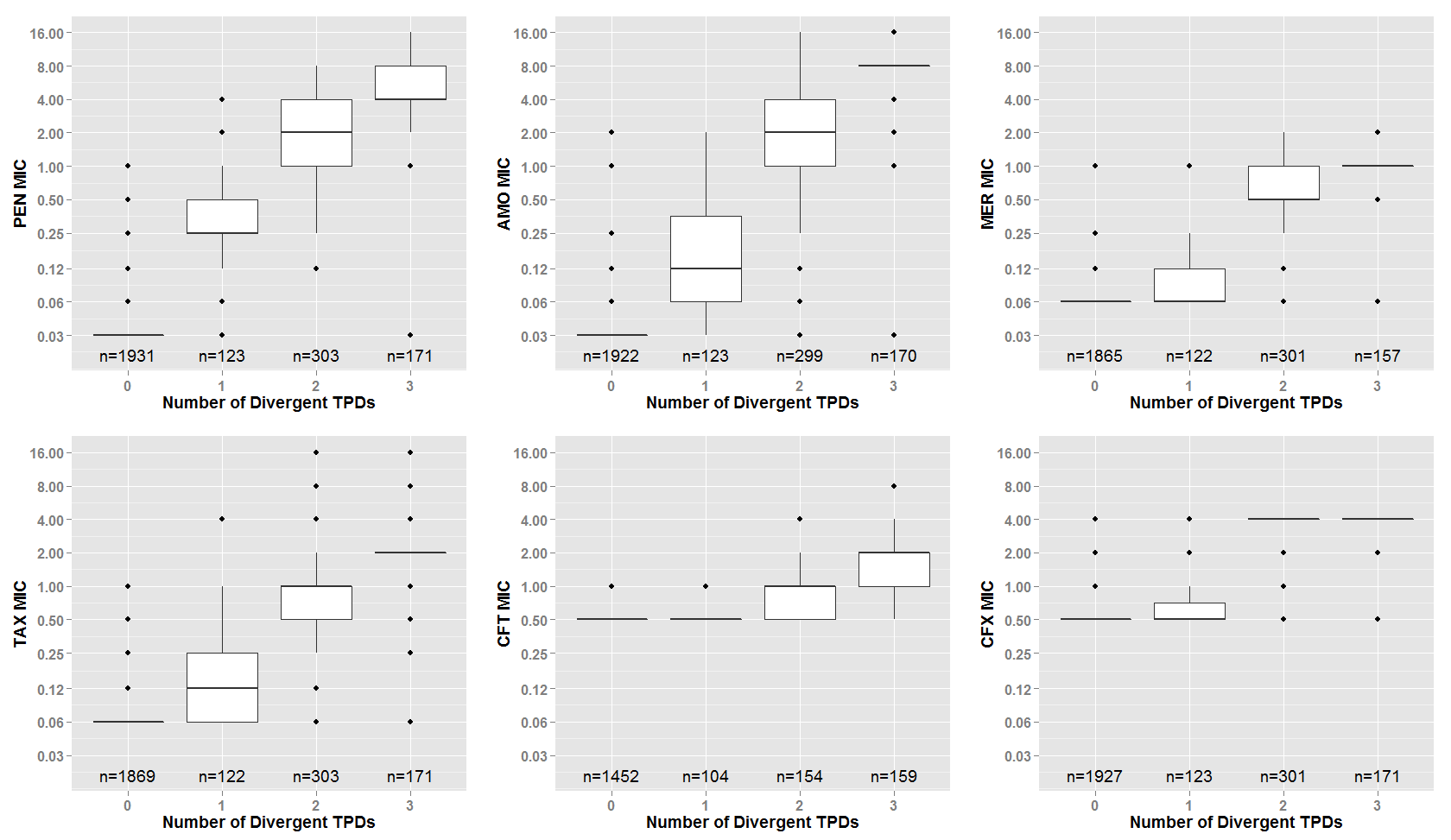

Supplement: Figure S2 — Boxplot of PEN MIC among isolates containing 0, 1, 2, or 3 divergent TPDs. A divergent TPD was defined as less than 90% amino acid sequence identity with the corresponding TPD in PBP type 2-0-2. Whiskers indicate the farthest value that is within 1.5 interquartile range (IQR) of the hinges. Download [file mbo004142843sf2.tif]

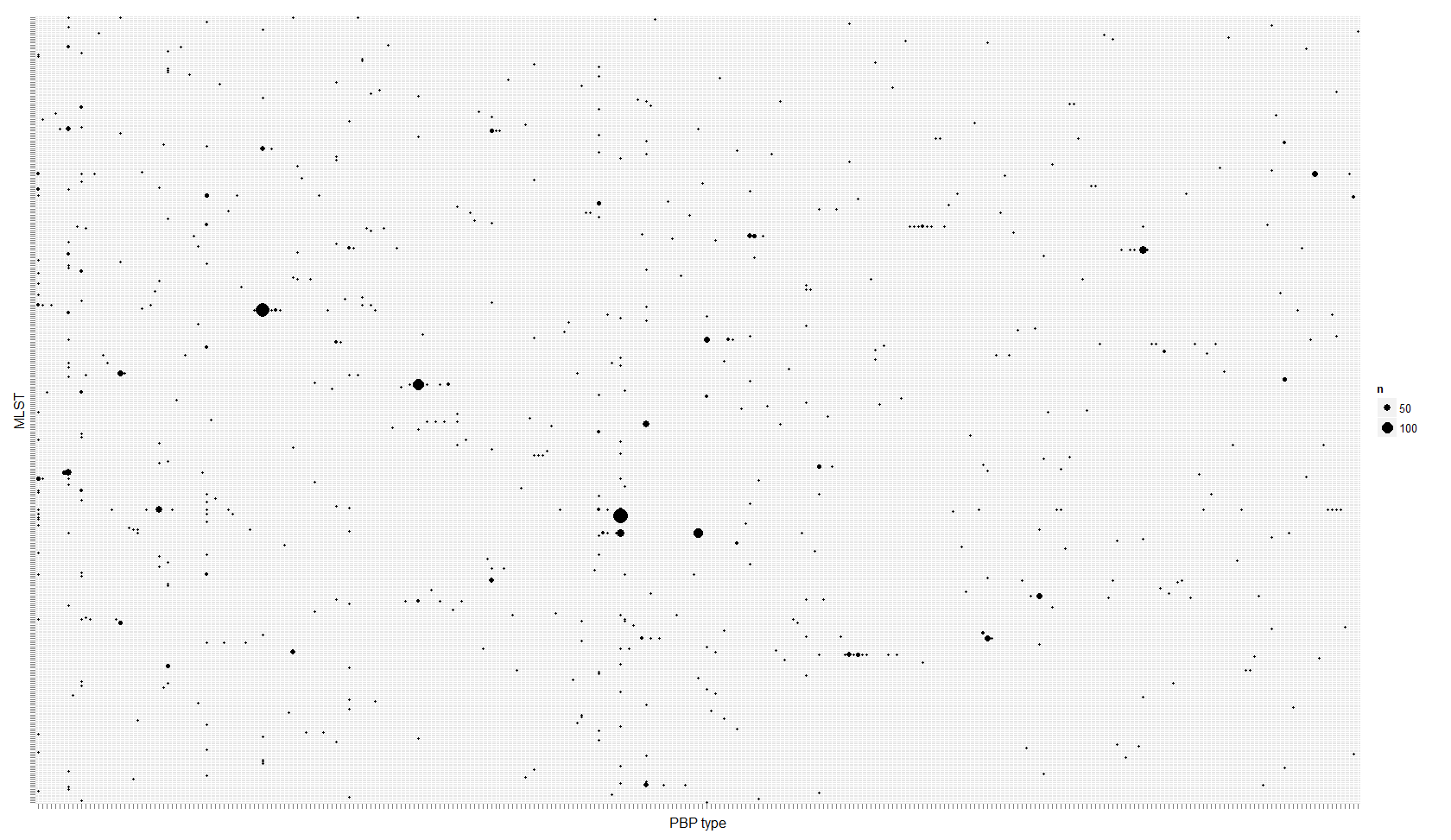

Supplement: Figure S3 — Distribution of isolates by PBP type and MLST. Each dot represents an isolate(s) with the same PBP type and MLST. The size of the dot is proportional to the number of isolates. Download [file mbo004142843sf3.tif]

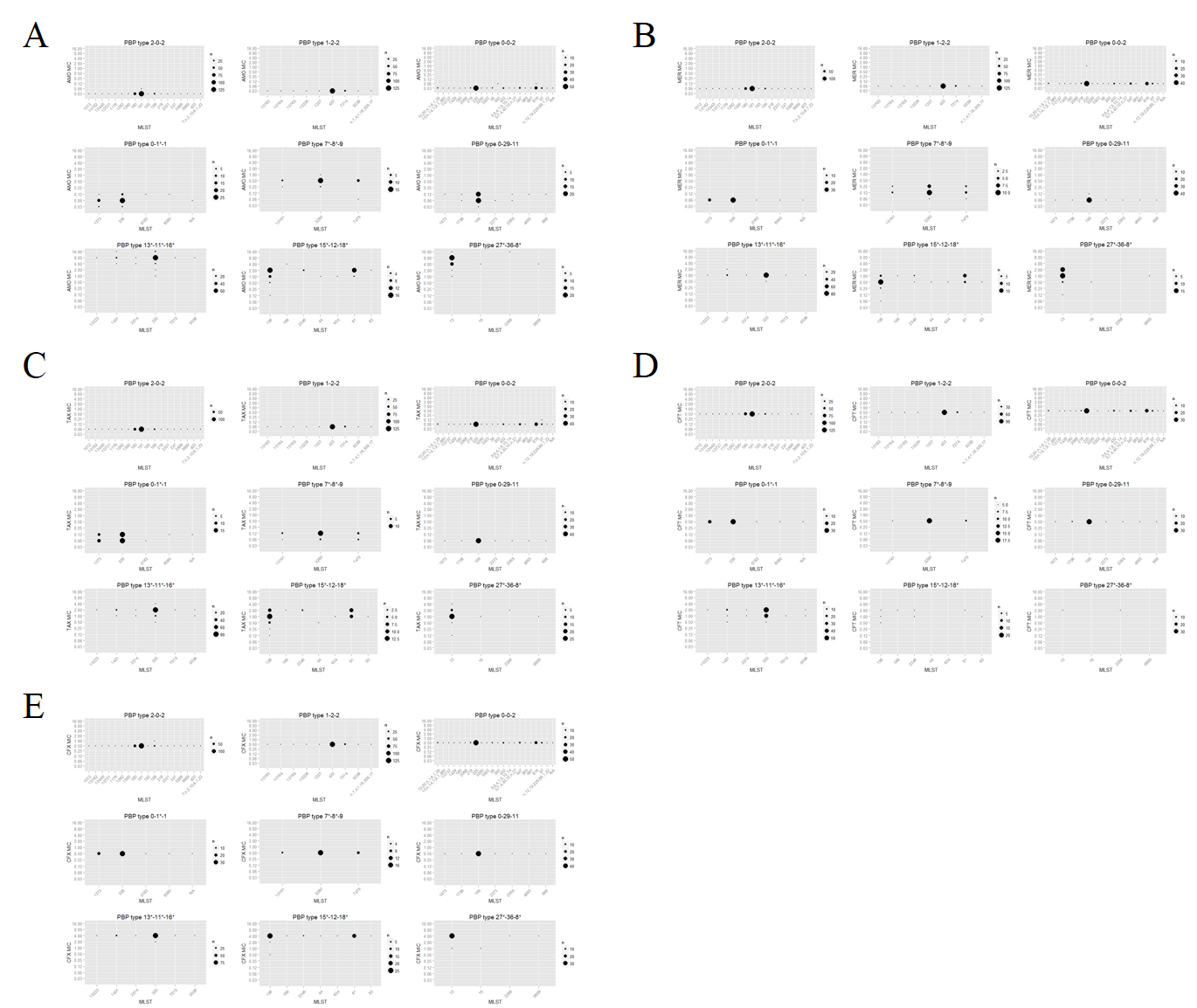

Supplement: Figure S4 — Distribution of MICs across MLSTs within nine representative PBP types for AMO (A), MER (B), TAX (C), CFT (D), and CFX (E). Download [file mbo004142843sf4.tif]

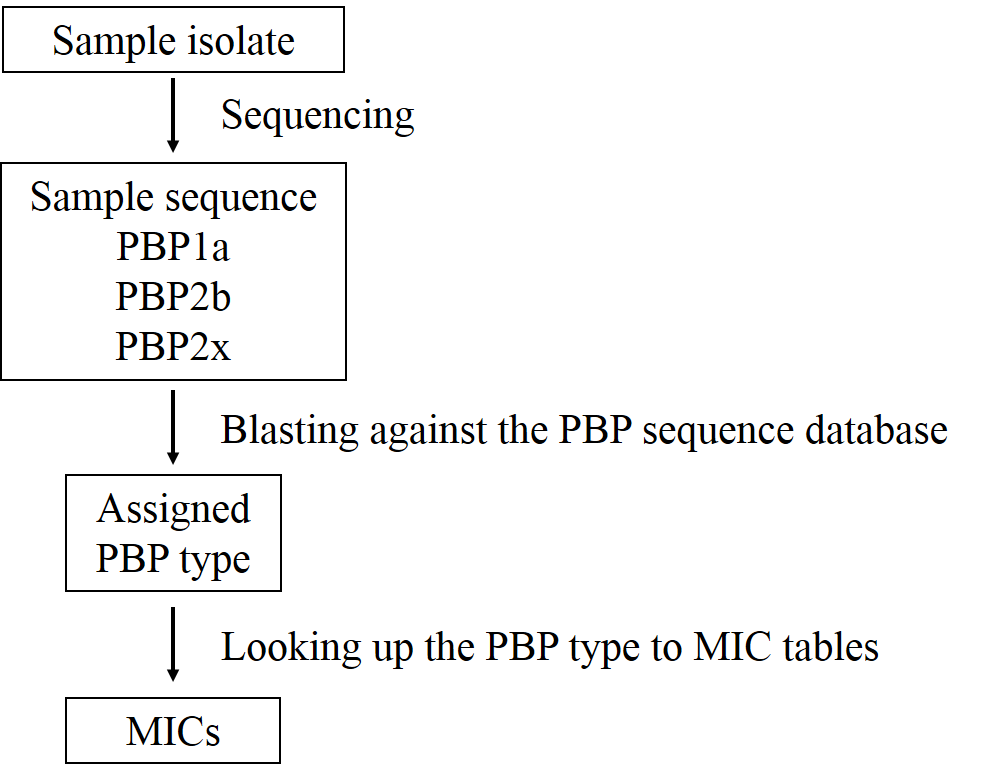

Supplement: Figure S5 — Diagram showing how to predict MICs for a specific isolate using the PBP sequence database (Table S4) and the PBP type to MIC tables (Table S2). Download [file mbo004142843sf5.tif]

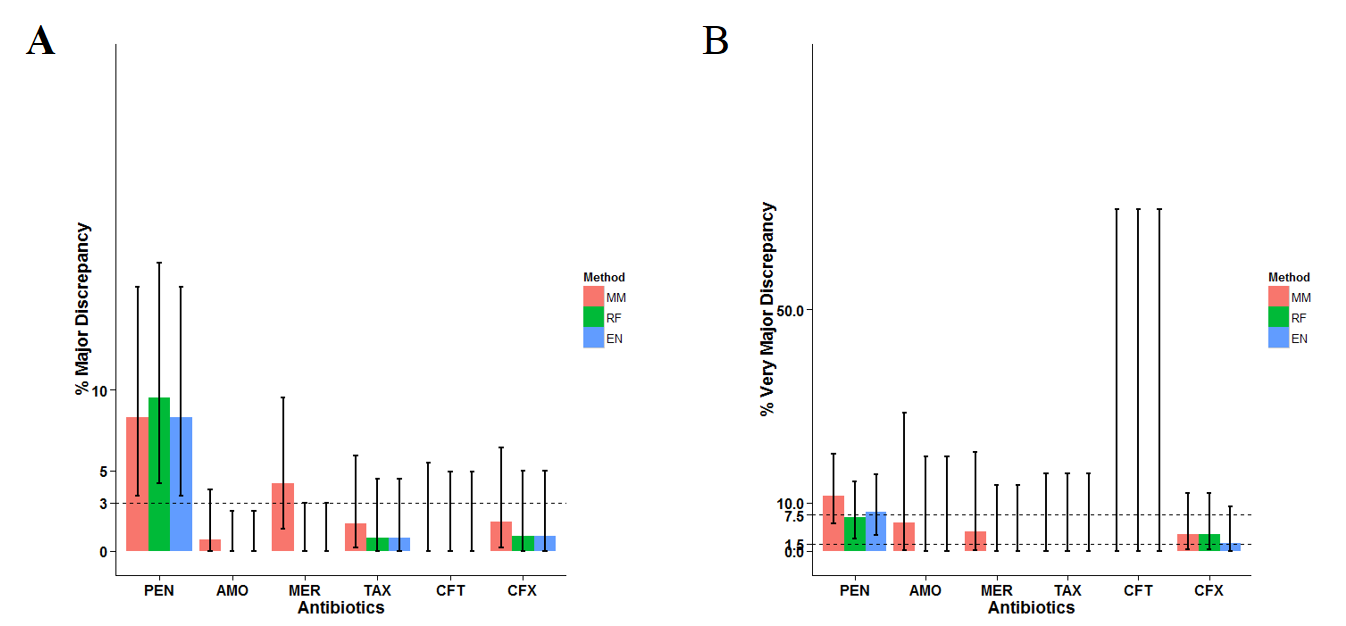

Supplement: Figure S6 — Agreement between the predicted MIC and the microdilution MIC among nontrained PBP types. See Materials and Methods for detailed model descriptions. (A and B) The rates of major discrepancy (A) and very major discrepancy (B) were calculated for the six antibiotics. Error bars are 95% confidence intervals. Download [file mbo004142843sf6.tif]
